# Live imaging and quantitation of nascent transcription using the MS2/MCP system in the *Drosophila* embryo

**DOI:** 10.1016/j.xpro.2021.100379

**Published:** 2021-03-18

**Authors:** Caroline Hoppe, Hilary L. Ashe

**Affiliations:** 1 Faculty of Biology, Medicine and Health, University of Manchester, Manchester M13 9PT, UK

**Keywords:** Microscopy, Model organisms, Molecular biology, Gene expression

## Abstract

Visualizing transcription live in *Drosophila* is providing important new insights into the spatiotemporal regulation of transcription. Here, we describe a protocol to visualize and quantitate transcription from gene loci that are tagged with MS2 stem-loop sequences in the *Drosophila* embryo. MS2 stem-loop sequences are recognized by a coat protein fused to a fluorescent protein and visualized with microscopy. We also describe an analysis pipeline to extract and subsequently quantify transcription dynamics.

For complete details on the use and execution of this protocol, please refer to Hoppe et al. (2020).

## Highlights

• Temporal transcription dynamics can be determined using MS2 live imaging
• *Drosophila* embryos are collected and mounted for live imaging
• Nascent transcription in the early embryo is detected by live confocal imaging
• An image analysis pipeline extracts the transcriptional profile for each nucleus

## Graphical abstract

**Figure:**
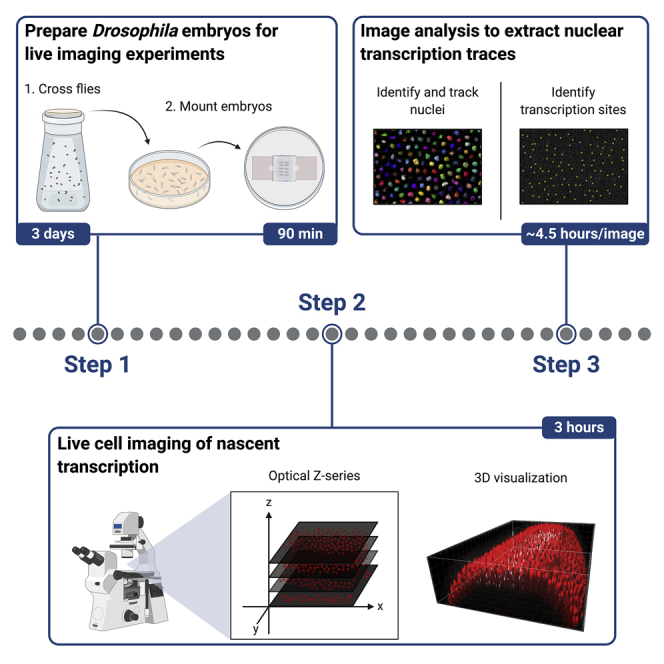

## Before you begin

### Experimental design considerations

**Figure 1:**
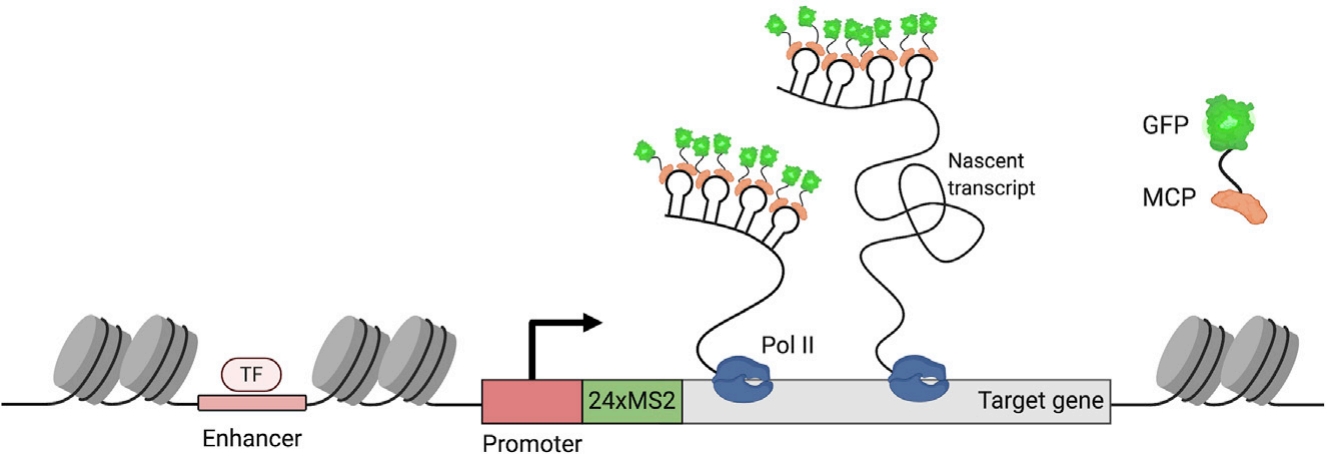
Visualization of transcription with the MS2/MCP imaging system The MS2 live imaging system uses RNA stem-loop repeats, derived from the MS2 bacteriophage that are bound by the cognate MS2-coat protein (MCP) fused to a fluorescent protein. Transcription factor = TF.

**Figure 2:**
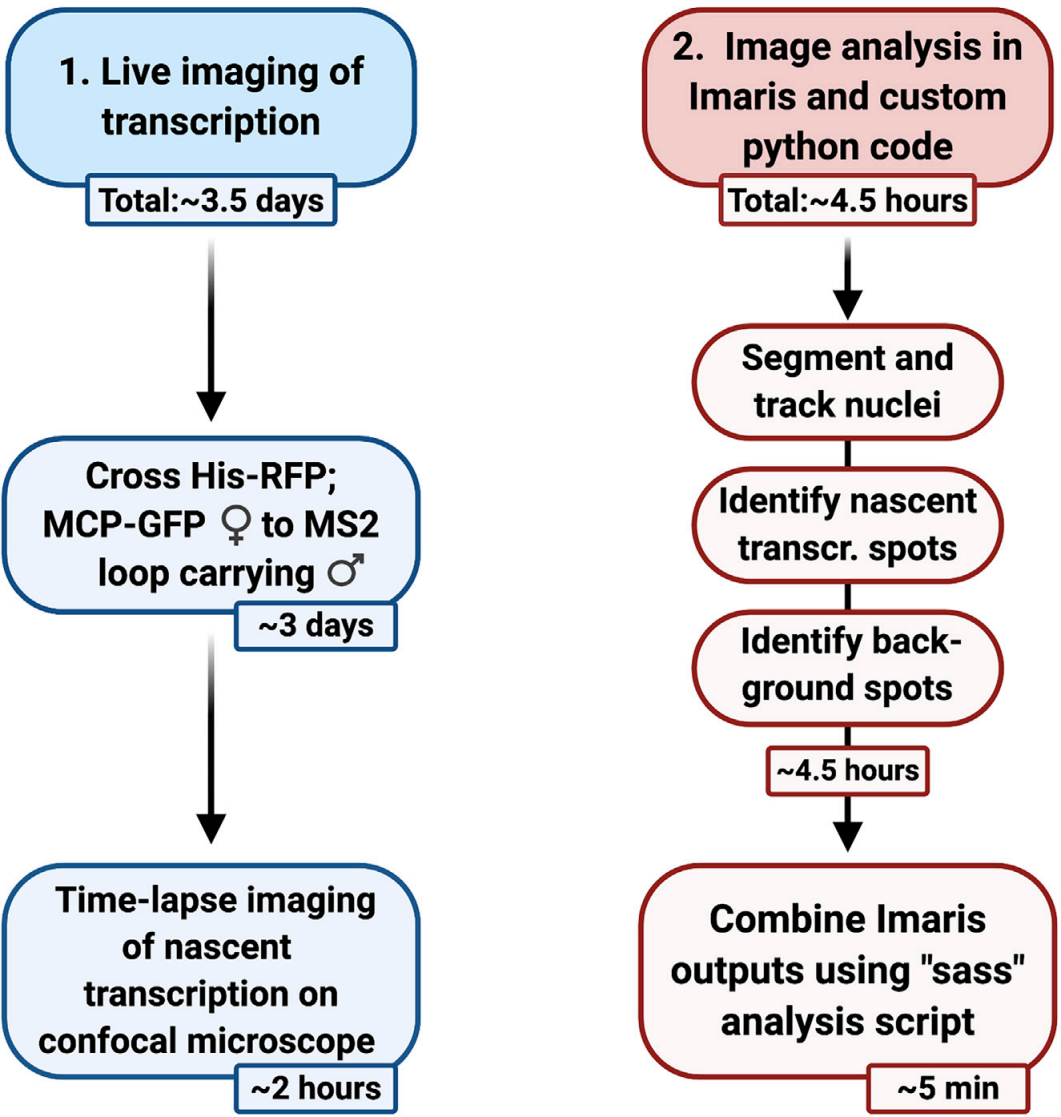
Overview showing image acquisition and image analysis covered in this protocol The steps are outlined with associated timings.

The MS2/MS2 coat protein (MCP) imaging system (Figure 1) has been used to extract transcription dynamics at the single-cell level with high spatial and temporal resolution in tissue culture cells and *in vivo* (Bertrand et al., 1998; Dufourt et al., 2018; Falo-Sanjuan et al., 2019; Fukaya et al., 2017; Fusco et al., 2003; Garcia et al., 2013; Hoppe et al., 2020; Koromila and Stathopoulos, 2019; Lee et al., 2019; Lionnet et al., 2011; Lucas et al., 2013; Scholes et al., 2019; Yamada et al., 2019). In *Drosophila* this technique has been used to investigate how target genes respond to different signals and transcription factor inputs (Berrocal et al., 2020; Bothma et al., 2014; Falo-Sanjuan et al., 2019; Garcia et al., 2013; Hoppe et al., 2020; Lammers et al., 2020; Lucas et al., 2013, 2018; Yamada et al., 2019), the function of enhancers and their contribution to transcriptional output (Bothma et al., 2015; Fukaya et al., 2016; Koromila and Stathopoulos, 2019; Scholes et al., 2019), transcriptional memory (Ferraro et al., 2016) and transvection (Lim et al., 2018a). The MS2 imaging system uses arrays of the MS2 bacteriophage derived stem-loop sequences that are inserted into non-coding regions of either endogenous genes (Fukaya, 2020; Hoppe et al., 2020; Lim et al., 2018b, 2018a) or transgenes (Falo-Sanjuan et al., 2019; Fukaya et al., 2017; Garcia et al., 2013; Lucas et al., 2013). Once transcribed by RNA Polymerase II (Pol II), the RNA MS2 stem-loop is recognized by its cognate coat protein MCP, which binds the loop structure as a dimer (Wu et al., 2012). Here, we describe how to mount embryos and image nascent transcription in living *Drosophila* embryos using confocal microscopy and how to quantify nascent transcription using an image analysis pipeline (Figure 2).

Visualization of nascent transcription is achieved by fusing the MCP protein to a fluorescent protein. The fluorescent signal intensity is influenced by the position of the MS2 stem-loops within the gene sequence. The signal will be brighter when imaging transcription if the MS2 stem-loops are inserted in the 5′ UTR (as shown in Figure 1) as opposed to their placement in the 3′ UTR. For the latter, MCP fluorescence will only become visible after Pol II has transcribed the 3′ UTR region, which is followed quickly by transcription termination and dissociation of the transcript from the nascent site of transcription, resulting in a weaker fluorescent signal (Garcia et al., 2013).

In addition, the fluorescence intensity is dependent on the number of MS2 stem-loop repeats inserted into the gene of interest. Most *Drosophila* studies use an MS2 stem-loop cassette containing 24 repeats, which has been shown to be sufficient to detect mRNA transcription dynamics (Falo-Sanjuan et al., 2019; Garcia et al., 2013; Hoppe et al., 2020; Lammers et al., 2020; Lucas et al., 2013). However, a stronger fluorescent signal is expected when imaging a gene with 128× MS2 stem-loops inserted (Tantale et al., 2016), which will facilitate the study of weakly transcribed genes.

*Drosophila* stocks are available that express MCP fused to different fluorescent proteins including GFP, RFP and mCherry. For example the following fly lines are available: P{nos-MCP-EGFP} [RRID:BDSC_60340], P{UAS-MCP-RFP} [RRID:BDSC_27419], P{nos-NLS-mCherry-MCP} (Bothma et al., 2018), P{nos-MCP-mCherry} (Liu et al., 2020) and P{nos-MCP-mCherry-FLAG} (Hayashi et al., 2014). During image analysis nuclear markers are used to identify and track nuclei through time, such as Histone2Av or Nucleoporin(107) fluorescent protein fusions (Garcia et al., 2013; Lucas et al., 2013).

***Note:*** A protocol outlining MS2 stem-loop insertion into transgenes and live imaging was published by Garcia and Gregor, 2018. A protocol describing how to insert MS2 stem-loop sequences into endogenous gene loci is described in the accompanying paper (Hoppe and Ashe, 2021).

***Note:*** In addition to the MS2/MCP system, an orthogonal mRNA tagging system, based on the *Pseudomonas* phage derived PP7 stem-loops and respective PCP coat protein, can be used to visualize nascent transcription (Chao et al., 2008; Pichon et al., 2018).

### Prepare heptane glue coated coverslips for the live imaging setup

**Timing: 10 min to coat coverslips; previously, soak the tape overnight (approximately 14 h) to remove glue from it**

The heptane glue coated coverslips will be used to position embryos for live-cell imaging.

1. Unroll sticky tape and transfer several lengths into a 50 mL reagent bottle.

***Note:*** Make sure that the sticky tape is not poisonous to *Drosophila* embryos. We use yellow double-sided plastic tape, 25 mm × 20 m (RS Components, Cat# 555-033) or packaging tape (Tesa, Cat# 4124).

2. Fill half of the bottle with heptane to dissolve the glue from the tape.
3. Keep the sticky tape in heptane for at least 14 h.
4. Pipette 60 μL viscous heptane glue onto the center of the coverslip (No. 1.5, 18 × 18 mm; Scientific Laboratory Supplies, Cat# MIC3120).
5. Let heptane evaporate in a fume hood until a thin film of glue is visible on the coverslip.

***Note:*** If the heptane glue is too viscous, dilute with more heptane. If you see debris on the coverslip during imaging, remove heptane glue from the sticky tape filled bottle, spin down in a microcentrifuge and transfer the supernatant to a new tube to use on new coverslips.

***Note:*** Make sure to protect the coverslips from dust and other dirt. For example, keep prepared coverslips in a clean Petri dish or another closed container.

***Note:*** Ideally prepare coverslips a few hours before the live imaging experiment. If stored in a closed container, coverslips can be kept for up to two weeks.

### Set up fly crosses for the live imaging experiment

**Timing: ∼3 days**

The purpose of this step is to set up fly crosses that are then used for embryo collection. Female flies of the genotype His-RFP; MCP-GFP (RRID:BDSC_60340) maternally deposit mature His-RFP and MCP-GFP fusion proteins into the egg. His-RFP is used to locate and track nuclei during imaging to aid analysis.

**Figure 3:**
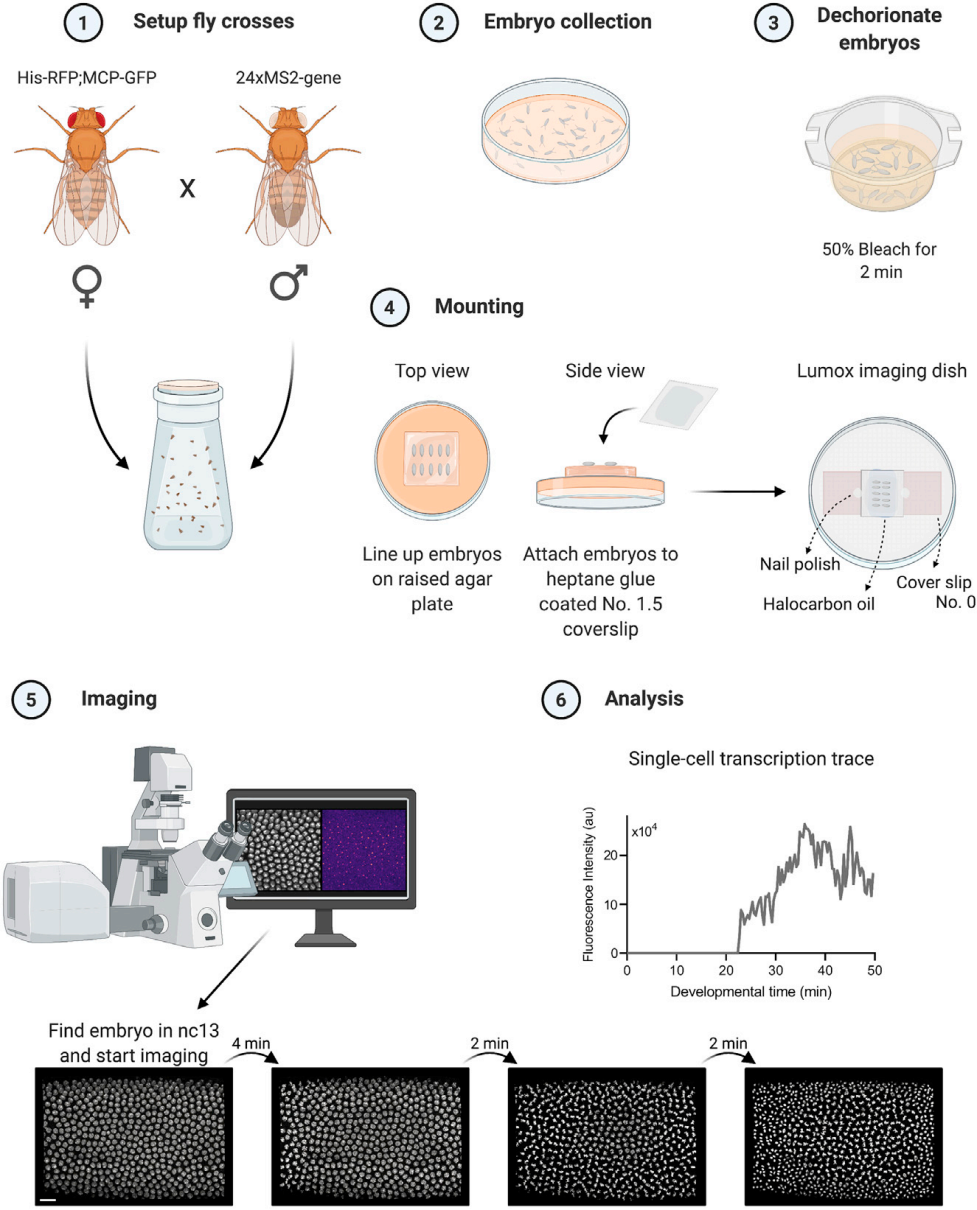
Overview of the live imaging experiment Female virgin flies (His-RFP; MCP-GFP) are crossed to homozygous males (24xMS2-gene) and kept in a collection bottle (1). Embryos are aged for 90 min and collected on apple juice agar plates (2) before embryos are dechorionated using 50% bleach (3). Dried embryos are lined up on a raised agar plate and attached to a heptane glue coated cover slip, which is added on top of a cover slip/halocarbon oil bridge (4). Embryo development is imaged on a confocal microscope (5), and single-cell fluorescent traces are extracted during subsequent analysis (6).

***Note:*** The protocol described here visualizes active transcription from one allele per nucleus.

***Note:*** Do not keep the fly cage for longer than one week as, in our experience, the incidence of developmental defects increases with age.

6. In order to obtain sufficient embryo numbers, a fly cage containing approximately 100 virgin females of the genotype His-RFP; MCP-GFP and at least 50 males carrying the MS2 loop locus is set up, ideally three days before the live imaging experiment and kept at 25°C (Figure 3, step 1). Males homozygous for the MS2 insertion should be used if possible.
  a. Cages are set up with apple juice agar plates containing yeast paste.

***Optional:*** In order to reduce the inherent background of unbound MCP-GFP, the overall concentration can be reduced by using females that are heterozygous for His-RFP; MCP-GFP. The amount of maternally deposited His-RFP and MCP-GFP protein will be reduced but still sufficient for imaging of transcription (Fukaya, 2020; Hoppe et al., 2020). This approach was used to acquire the example dataset supplied with this protocol.

***Alternatives:*** To image both gene alleles at the same time, His-RFP; MCP-GFP flies can be crossed first to homozygous MS2 flies and the resulting female offspring can be crossed again to males carrying the MS2 locus. Half of the resulting embryos will be homozygous for the imaging locus if the tagged locus is located on an autosome or a quarter for a gene on the X-chromosome.

## Key resources table

**Table:** 

REAGENT or RESOURCE	SOURCE	IDENTIFIER
**Chemicals, peptides, and recombinant proteins**		

Halocarbon oil 27	Sigma	Cat# H8773; CAS: 9002-83-9
Halocarbon oil 700	Sigma	Cat# H8898; CAS: 9002-83-9
2Work thin bleach	Banner	Cat# 1190012
TritonX-100	Sigma	Cat# X100; CAS: 9002-93-1

**Deposited data**		

Example datasets and analysis files	This study	Mendeley Data DOI: 10.17632/jv9fjpn9cg.1

**Experimental models: organisms/strains**		

*D. melanogaster*; *y*^*1*^*w∗*; P{w+mC=His2Av-mRFP1}; P{w+mC=nos-MCP.EGFP}	Bloomington Drosophila Stock Center	RRID:BDSC_60340
*D. melanogaster*; *y*^*1*^*w*^*67c23*^*; 24xMS2-ush*	Hoppe et al., 2020	N/A

**Software and algorithms**		

Imaris 9.2	Bitplane	RRID:SCR_007370
LAS X	Leica Microsystems Inc	https://www.cellularimaging.nl/leica-las-x/
Code combining single-cell traces and spot detection from live imaging movies and using Imaris output	Hoppe et al., 2020	https://github.com/TMinchington/sass, RRID:SCR_018797

**Other**		

Leica TCS SP8 AOBS inverted microscope	Leica	N/A
Lumox dish 50, cell culture dish	Sarstedt AG & Co	Cat# 94.6077.305
Coverslips no. 1.5, 18 × 18 mm	Scientific Laboratory Supplies	Cat# MIC3120
Coverslips no. 0, 18 × 18 mm	Scientific Laboratory Supplies	Cat# MIC3100
Yellow double-sided plastic tape	RS Components	Cat# 555-033
Packaging tape	Tesa	Cat# 4124

## Step-by-step method details

### Embryo collection and mounting

**Timing: Embryo collection, ∼90 min; mounting, ∼10 min**

This section describes how embryos are collected and mounted for live-cell microscopy.

1. On the morning of the experiment, change the fly cage plate to a fresh apple juice agar plate containing yeast paste and wait 1 h, to give the flies time to lay retained embryos. After 1 h change the plate again. If the gene of interest is expressed during nuclear cycle (nc) 14, collect embryos for 90 mins (Figure 3, step 2).
2. Embryos are washed off the plate with NaCl (0.7%)/TritonX-100 (0.05%; Sigma, Cat# X100) wash solution and dechorionated by adding freshly prepared 50% bleach (Banner, Cat# 1190012) to the plate for 2 min. Next, decant embryos into a sieve and wash them with water until they are free of bleach (Figure 3, step 3). Dry the sieve with paper towels and remove as much water as possible from the embryos.
3. Use a blade or tweezers to cut a square from a fresh apple juice plate, turn the plate over, and place the square on top of the plate so it is elevated. Move the plate under a dissection microscope. Transfer embryos onto the agar square using a brush (Figure 3, step 4).
4. Line up the embryos and orient them so that the area in which the gene of interest will be expressed is facing upwards (Methods video S1).

***Note:*** Position the embryos in one or two lines to make navigation under the microscope easier.

Methods video S1. Mounting *Drosophila* embryos for live imaging experiments, related to step 4

Mounting approximately 20 embryos will ensure that multiple embryos are at the correct developmental stage for imaging.

***Note:*** Try to exclude embryos that have already entered late nuclear cycles or are gastrulating. These embryos appear to have a transparent periphery and a more dense and structured center (Methods video S1).

5. Next, prepare a cover slip/halocarbon oil bridge on a Lumox imaging dish (Sarstedt AG & Co, Cat# 94.6077.305) (Figure 3, step 4).
  a. Use a cut pipette tip to add 70 μL of halocarbon oil (7:1, halocarbon oil 700:halocarbon oil 27; Sigma Cat# H8898 and Cat# H8773) onto the middle of the imaging dish.
  b. On each side of the drop of oil place a coverslip (no. 0, 18 × 18 mm; Scientific Laboratory Supplies, Cat# MIC3100) and create a bridge (see Methods video S1 and Figure 3, step 4). The two coverslips should be approximately 0.6 cm apart.
    i. No. 0 cover slips used in this setup have a thickness of approximately 100 μm (Scientific Laboratory Supplies), whereas a *Drosophila* embryo has an approximate height of 180 μm (Markow et al., 2009). Therefore, the embryo becomes flattened against the coverslip.

***Note:*** Prepare one Lumox imaging dish per experiment. The dishes have a gas-permeable membrane and can be reused a few times before the membrane becomes too flexible (see troubleshooting 1).

6. Fix embryos in place with a heptane glue coated coverslip (Figure 3, step 4).
  a. Carefully pick up the heptane glue coated coverslip and gently drop it onto the embryos with the heptane glue side facing down toward the embryos.
  b. Use tweezers to (very carefully) press the coverslip onto the embryos. You will notice that the embryos flatten slightly and press against the coverslip.
  c. Pick up the coverslip with the embryos attached to it and place it onto the coverslip-bridge, sandwiching the embryos between the dish membrane and the coverslip in the drop of oil. Secure the top coverslip in place with a dot of nail polish.

**CRITICAL:** If the embryos are not attached properly to the heptane glue coated coverslip, they might start sliding during the live imaging time lapse. If embryos are flattened too much by the heptane glue coated coverslip, they may arrest during a mitotic cleavage cycle or display mitotic defects (see troubleshooting 1).

### Live-cell microscopy of nascent transcription

**Timing: 2 h**

In this section, the microscopy setup and settings are outlined to visualize nascent transcription. The prepared sample can be imaged on an upright or inverted microscope setup. Here, embryos were imaged on an inverted Leica SP8 confocal microscope (Figure 3, step 5).

7. For imaging experiments, we use a Leica TCS SP8 AOBS inverted confocal microscope with a resonant scan head and a 40×/ 1.3 HC PL apochromatic oil objective with the pinhole set to 1.3 airy units.
  a. The microscope settings used to image the sample dataset were as follows:
    i. The scanning frequency of the resonance head was 8,000 Hz (with a minimum zoom of 1.25).
    ii. Scanning was set to bidirectional.
    iii. Images were acquired at 8 bit and with 1,024 pixels per line and 700 lines. These settings result in a pixel size of 378.79 nm × 378.79 nm, an image size of 387.5 μm × 264.77 μm and a pixel dwell time of 1.04 μs. This setup allows us to capture most of the *Drosophila* embryo.
  b. Images were collected using hybrid detectors and fluorophores were excited with a white laser. The example dataset was acquired using the following settings:
    i. White laser set to 70% with 488 nm (8%), 561 nm (2%) and 8× line averaging. These settings result in the following background corrected laser powers: 6.34 μW (488 nm) and 2.88 μW (561 nm). Ensure minimal photobleaching (see troubleshooting 2) and that the signal is not saturated.
  c. Z-stacks were acquired continuously with 1 μm steps. A depth of 55 μm per z-stack can be achieved with a final temporal resolution of 20 s per time frame.
    i. If the gene of interest is not highly expressed, the z-step size can be reduced to ensure that the transcription site is imaged in multiple z slices.
8. Find embryos on the imaging dish using brightfield illumination and rotate to align the embryos horizontally with their AP axis.
9. Check the age of embryos by looking at the nuclei using fluorescence illumination in the 561 nm excitation line. Pick an embryo where the nuclei have migrated to the surface and are currently undergoing nc13 (Figure 3, step 5).

10. Set up a time lapse and include the full depth of nuclei in the z-direction. A maximum projected movie of the example time-lapse experiment can be found in Methods video S2 (previously described in Hoppe et al., 2020).
  a. It is advisable to set up the z-stack to include a number of slices below the nuclear volume at the start of imaging. This extra depth ensures that the nascent transcription sites are fully included within the imaging volume while nuclei elongate during nc14 (see example datasets).
    ***Optional:*** Deconvolve the time-lapse data using the inbuilt Leica lightning deconvolution software. As an alternative, the Huygens Professional Deconvolution software can be used to deconvolve images but struggles with large file sizes.
    **CRITICAL:** When imaging make sure that the full fluorescence of the transcription sites is included in the z-stack (not cut off on the top or bottom). If the expression domain of the gene of interest is very wide, imaging is potentially complicated by the degree of embryo flattening. In our experience, an active transcription site is visible in approximately 6–8 z slices at its maximum transcriptional activity.
    ***Note:*** Depending on the expression level, the fluorescence might be very weak and the imaging/genomic setup will need to be optimized (see troubleshooting 3).
    ***Note:*** If a different microscope is used the most important setting to consider is the imaging speed. As transcription is a very dynamic progress, care should be taken to accommodate a high frame rate. The time resolution of 20 s, as described in this protocol, can be improved further by imaging a smaller region of the embryo or smaller z-stacks. Achieving a high time resolution is often associated with a tradeoff in the image quality. Low raw image quality can complicate image analysis and potentially mask real signal under the background haze. Therefore, we recommend to use a microscope with a resonant scanner, a Piezo stage fixture, a Piezo objective collar, or alternative options to ensure high speed imaging.
  Methods video S2. Maximum intensity projection of time-lapse experiment showing transcription of the endogenous *ush* gene using the MS2/MCP system, related to step 10 Time-lapse video was taken on an inverted SP8 confocal microscope (40× objective, depth of 55 μm and a final temporal resolution of 20 s per time frame).

### Tracking of nuclei and identification of transcription sites

**Timing: ∼ 4.5 h/time-lapse dataset**

This next section describes the initial semi-automated data analysis pipeline to extract the fluorescent signal from each nucleus and track the signal over time (Figure 3, step 6). This pipeline combines data analysis performed using Imaris software 9.2 (Bitplane, Oxford Instruments, Concord MA, RRID:SCR_007370) with open-access, custom python scripts. A sample time-lapse dataset (Embryo1_full_analysis.ims) with full nuclear traces and the corresponding analysis files is provided with this protocol. Additionally, a beginners dataset containing a small number of nuclei, time points and the corresponding analysis files is provided.

Files are deposited at Mendeley Data: https://doi.org/10.17632/jv9fjpn9cg.1

***Note:*** This analysis pipeline was conceptualized to be easily applicable to time-lapse datasets acquired using different microscopes, magnifications and of different embryo regions. In addition, this pipeline does not require coding knowledge or familiarity with MATLAB/python or other programming languages. A similar Imaris based pipeline has been developed independently by Yamada et al (2019).

**CRITICAL:** The time it takes to analyze an embryo dataset varies significantly with the quality of raw data, the size of the field of view and the number of time frames. The example dataset includes a cropped field of view containing approximately. 116 nuclei. The beginners dataset provides approximately 20 nuclei for quicker analysis using this pipeline.

***Alternatives:*** Other custom software is available to track nuclei and quantitate nascent transcription. Custom MATLAB scripts have been generated by Garcia et al., 2013 and further optimized by Lammers et al., 2020. MitoTrack is an open-access software that can track nuclei and extract MCP fluorescence from maximum projected images, while keeping a lineage tree history through cell divisions (Trullo et al., 2020).

***Note:*** We outline the rationale of each analysis step to enable users of other analysis platforms to incorporate some aspects of this pipeline.

**Figure 4:**
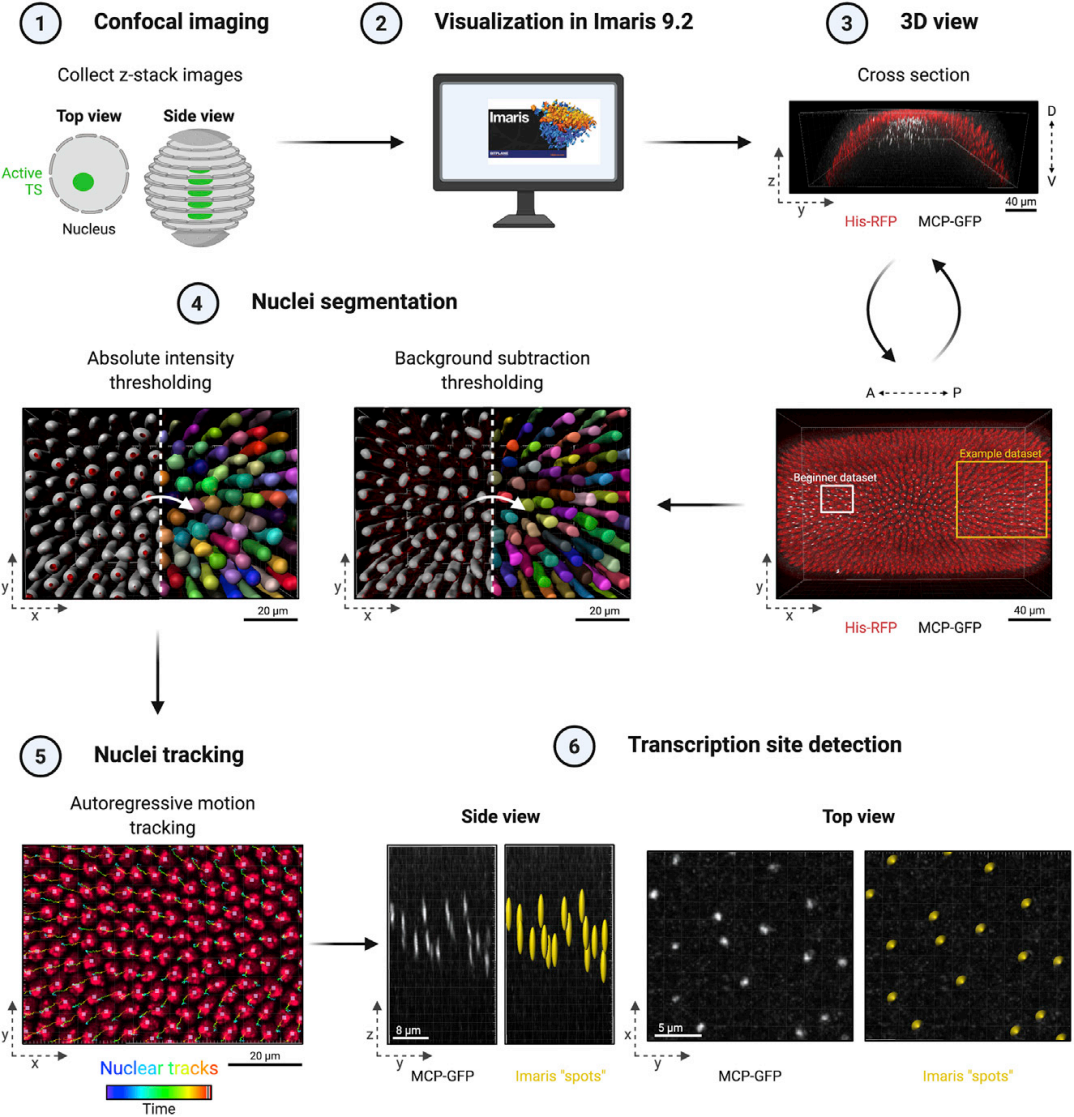
Semi-automated analysis pipeline The developing *Drosophila* embryo is imaged over time using z-slices to cover the full depth of the nucleus and capture the full fluorescence (1). Images can be analyzed in the Imaris software (2), which allows the images to be rendered in 3D (3). The time-lapse dataset is cropped to a region of interest for further analysis. Nuclei are segmented using the “surfaces” module, the His-RFP channel and either “absolute intensity” or “background subtraction” thresholding (4), before they are tracked through time using an autoregressive motion algorithm (5). Transcription sites are segmented using the “spots” function and the MCP-GFP channel (6).

***Note:*** Imaris supports all common microscopy file formats.

11. Load time-lapse microscopy data into Imaris 9.2 to render images in 3D (Figure 4, steps 1–3).

**Nuclear segmentation:** As a first step, nuclei are segmented by creating a surface over objects detected in the His-RFP channel. In order to ease segmentation, the detectable objects are smoothed using a Gaussian filter (“smooth surface detail” setting).

12. Identify and segment nuclei based on the His-RFP fluorescence channel and using the inbuilt Imaris “surfaces” function. Follow the algorithm steps as they are displayed in the software.
  a. Select “track surfaces over time” in algorithm settings and either analyze the entire image or select a region of interest.
  b. In the next step, select the His-RFP channel as the source channel and set “smooth surface detail” to 1 μm.
  c. Different thresholding options can work well to segment nuclei.
    i. Absolute intensity thresholding option: When choosing “absolute intensity” select “split touching objects” to prevent the fusion of multiple nuclei. Defining the seed point diameter as 3 μm is a good starting point (Figure 4, step 4).
    ii. Background subtraction thresholding option: When choosing the “background subtraction” thresholding method, choose 1.3 μm as a start value for “diameter of largest sphere which fits into object.” In the next step, move the slider until nuclei are sufficiently identified and shaded (Figure 4, step 4). In our experience, it is easiest to make sure that the shaded spheres do not touch each other, instead of using the seed point option, to prevent any fusion to form one nucleus in subsequent steps.
      ***Note:*** Nuclei in the example dataset were segmented using the “background subtraction” thresholding. Examples of both thresholding methods for one time point are included in the example dataset and were used to generate the images in Figure 4, step 4.
  d. In the next window, choose classifiers to separate nuclei by selecting as many filters as needed to obtain the best segmentation result. Filters can help to remove false-positive results from the segmentation. We find the following filters especially helpful: Number of voxels, Position Z, Distance to image border XY, Quality and Area.
    **CRITICAL:** Achieving a good segmentation result is important to prevent false-positive/-negative results during later analysis steps.
    **Nuclear tracking**: Imaris offers a number of different tracking algorithms that predict the surface (nuclear) object positions in the next frame. Based on the predicted positions the best matches are selected using a linear assignment algorithm to form track connections. The autoregressive motion tracking option considers the previous time point and predicts the same amount of object movement in the next time frame. The user can determine the distance an object is allowed to deviate from the predicted path (“maximum travel distance” input). More detailed information can be found in the Imaris handbook.
  e. Nuclei are tracked through time in 3D using the inbuilt autoregressive motion algorithm. The maximum frame gap size is set to 5 and the maximum travel distance is set to 5 μm (Figure 4, step 5).
  f. After tracking is complete, tracks and nuclei can be corrected manually in the “edit” and “edit tracks” tabs.
    ***Alternatives:*** Segmentation accuracy may vary based on raw data quality. Sometimes it can be difficult to find segmentation settings that will work on the whole time-lapse dataset. This is especially apparent if nuclei change shape or photobleaching has occurred. In this case, it can be easier to disable “track surfaces over time” in the surface wizard and segment nuclei in smaller time frame groups. Segment 10–50 time frames at a time by selecting them in the “region of interest” window. Follow the segmentation wizard to the end. Then, merge nuclei groups by selecting “merge” in the surfaces editing tab. After merging all nuclei, select “tracking” in the “rebuild” tab to track nuclei through time. Using this approach, nuclei segmentation can be manually adjusted and corrected before tracking. Nuclei in the example dataset were segmented in sets of 40 time frames, then merged and tracked.

**Transcription site identification:** To determine the transcription activity, we quantify the full (sum) fluorescence of nascent transcription sites in 3D. This is achieved by placing spheres around the transcription sites that encapsulate the full fluorescence, which can then be measured.

13. Active transcription sites are identified in the MCP-GFP channel, using the inbuilt Imaris "spots" function. Follow the steps of the creation wizard.
  a. Analyze the full image or choose a region of interest. Select the MCP-GFP channel as “source channel” to identify nascent transcription foci.
  b. In the next window, specify the spot/sphere size.
    i. Transcription foci can be estimated to be 1.8 μm in XY diameter.
    ii. The “model PSF-elongation along z-axis” will differ based on the microscope that was used and needs to be determined based on the dataset to be analyzed.
  c. In the next step, three-dimensional spheres, of identical size, are placed around the transcription signal by the software (Figure 4, step 6). It is important that the full fluorescence is contained within the sphere.
    i. When analyzing a new genotype, check that the full fluorescence is contained within the sphere at time points where the fluorescent signal is brightest.
    ii. If this is not the case, return to the previous step in the creation wizard and adjust the sphere size.
  d. Next, filters are used to classify spots. Select as many filters as needed to remove false-positive spots and identify as many “real” fluorescence spots as possible. We find the following filters especially helpful: Quality, Sum Intensity of MCP-GFP channel, Position Z, and Distance to image border XY.
    i. False-positive spots are most often detected outside the z-plane of transcription sites with a lower sum fluorescence than the average transcription site.
  e. Before continuing, check the spots that were created by the software by navigating through the time frames. When the fluorescent signal is very dim, transcription sites are sometimes not detected (see troubleshooting 4) or false-positive spots are added (see troubleshooting 5).

***Note:*** To change the size of all spot objects at the same time, a python based Imaris X-Tension “XTSpotResizer” is available. This X-Tension, written by Egor Zindy, can be downloaded at https://github.com/zindy/Imaris and integrated into Imaris under Edit -> Preferences -> Custom Tools. Locate the folder containing the downloaded “SpotResizeDialog.py,” “XTSpotReziser.py,” “TkDialog.py,” and “BridgeLib.py” in the X-Tension folder field and add it to the custom tools. The X-tension will appear in the Image Processing menu.

***Note:*** In the example dataset, transcription sites were excluded from nuclei sitting on the edge of the field of view as the onset of transcription could have been missed or the nascent transcription sites are not fully present in the field of view.

**Background correction:** The inherent background fluorescence, originating from unbound MCP-GFP, is removed using the sass.py script. A linear regression line is fitted based on background fluorescence values determined in this step. The line equation will then be used to determine the average background fluorescence present at each time point and subtracted from the transcription site fluorescence measurements.

**Figure 5:**
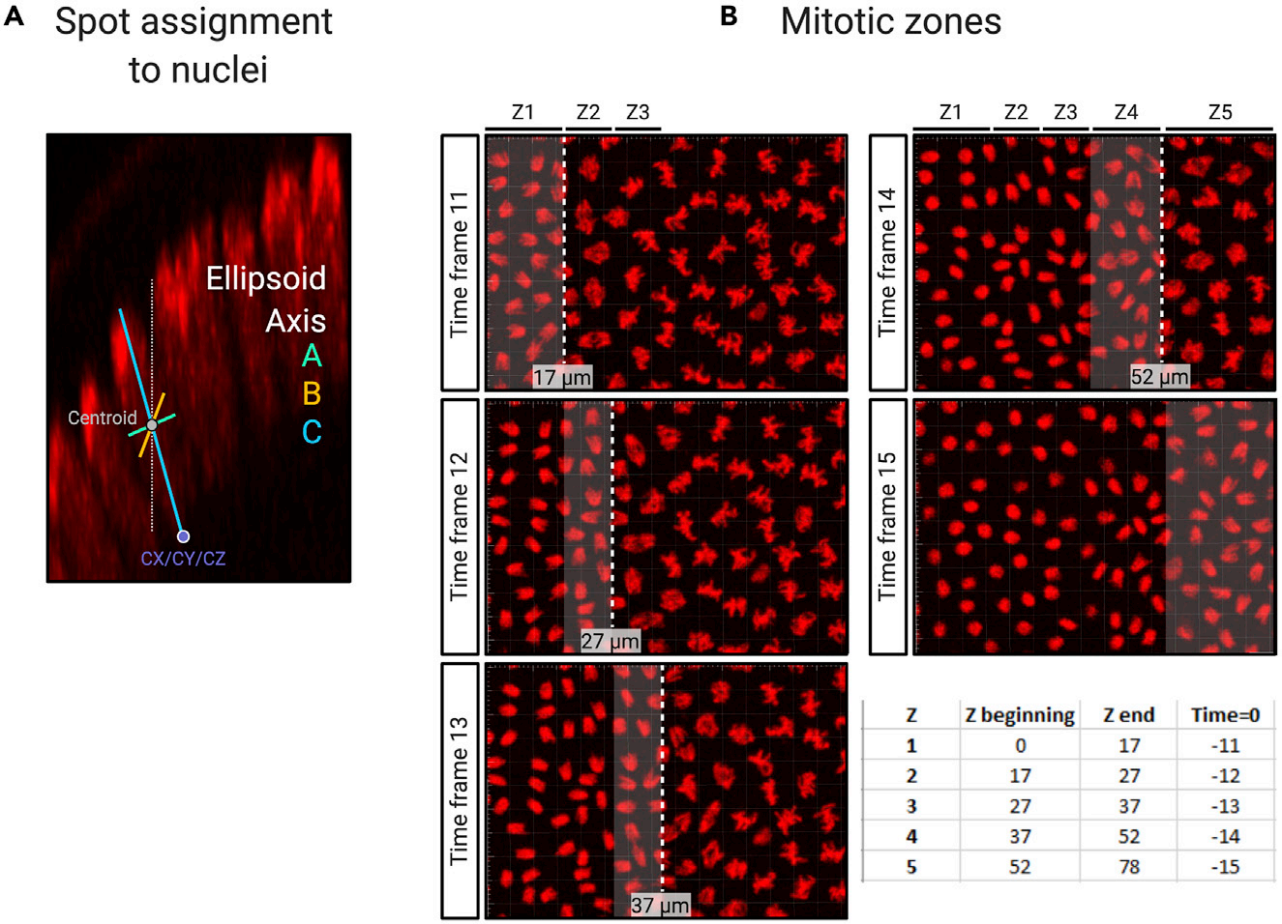
Spot assignment and mitotic wave correction (A) Each nascent transcription site is assigned to the closest nucleus in three dimensions using the proximity to the nuclear ellipsoid axis C (blue). Use of the ellipsoid axis C instead of a perpendicular axis (white line) results in a more accurate spot assignment in curved regions of the embryo. (B) Time differences arising from the mitotic wave are corrected during the analysis. The expression domain is separated into mitotic zones that contain nuclei that undergo telophase in the same frame. The x-axis positions of the zone borders are recorded together with the number of time frames that need to be adjusted in order to reset t = 0.
  c. Under File -> Preferences -> Statistics -> Spots make sure that at least “Intensity Sum” and “Position X/Y/Z” are selected. Add other desired fluorescent measurements.
  d. Select the surface icon that contains the tracked nuclei. Select the “statistics” tab and “detailed.” Export “all statistics to file.” Make sure the filename contains the word “cells” (Figure 6, step 1). This generates a folder containing an excel sheet for each statistic.

14. Manually add a second set of spots, of the same size as spots used to identify transcription foci, to generate background readings.
  a. Identify the first time frame that contains an MS2 signal. Add “new spots” using the Imaris wizard. On the first wizard tab, select “skip automatic creation, edit manually.” Manually add 4 spots (pointer in the “select” mode, press shift and left click) in the nuclear layer but outside of the expression domain or away from nuclei with active transcription. Make sure the background spots have the same size as spots used to identify transcription foci.
  b. Add 4 background spots in every third-fifth time frame until the end of the time-lapse movie. We find these time intervals to be sufficient to create an accurate linear regression line.
15. Export data files:
  a. Under File -> Preferences -> Statistics -> Surfaces make sure that at a minimum “Position X/Y/Z,” “Ellipsoid Axis CX/CY/CZ” and “Ellipsoid Axis length C” are selected.
  b. The “Position” output file will record all nuclear centroid X/Y/Z positions, nuclear ID and TrackID for every time frame. The Ellipsoid Axis C X/Y/Z positions and length determines the orientation of the ellipsoid nuclear axis in the C direction (Figure 5A).

**Figure 6:**
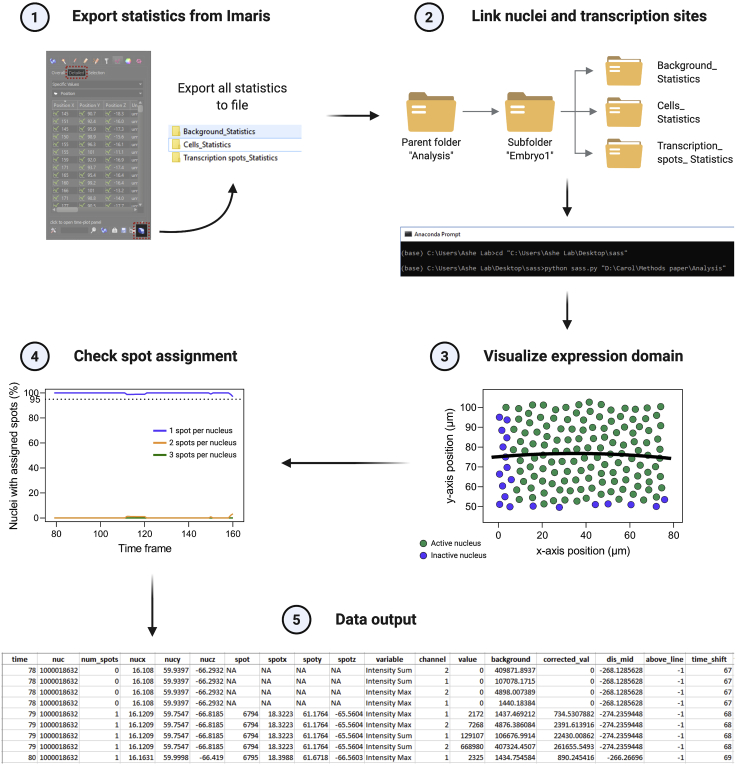
Combining the Imaris output files of nuclei and spots using a custom python script The statistic files of spots and surfaces are exported from the Imaris software (1) and saved in a subfolder. Using the custom python script “sass,” each detected transcription site is assigned to the closest nucleus (2). “Sass” outputs multiple images to investigate the analysis accuracy including plots of the expression domain (3) and the percent of nuclei that were wrongly assigned 2 or 3 spots (4). All the data are outputted in a large data file (5).
  e. Select the spots icon that contains the identified transcription sites. Select the “statistics” tab and select “detailed.” Export “all statistics to file.” Make sure the filename contains the word “spots.”
  f. Select the spots icon that contains generated background spots. In the “statistics” tab select “detailed.” Export “all statistics to file.” Make sure the filename contains the word “background.”

**CRITICAL:** A specific folder structure is needed to run the custom python script: parent folder -> subfolder containing Imaris statistics. The example dataset contains the following structure: Analysis -> Embryo 1 (Figure 6, step 2).

***Optional:****Drosophila* embryos undergo rapid mitotic divisions during early development. After nuclei have migrated to the embryo periphery, mitotic divisions occur in a wave (Foe, 1989; Foe and Alberts, 1983). The time differences in transcriptional onset that result from the mitotic wave can be corrected using the following pipeline. The temporal profile of cells is synchronized using the file “key_time.txt” contained within the sample analysis file. In this file, manually note the x-axis positions (Imaris slice view, increasing numbers from left to right) of boundaries that contain a group of similarly timed nuclei (columns 1 and 2) and in column 3 note down the time frame in which nuclei undergo telophase. During subsequent data analysis, time frames are adjusted by the calculated time correction using the telophase frame number within mitotic zones (Figure 5B).

### Assigning transcription sites to nuclei

**Timing: ∼10 min/time-lapse dataset**

**Assign transcription sites to nuclei:** The custom python script uses the Ellipsoid axis to assign spots to nuclei instead of the centroid position. This helps to assign each transcription site to the correct nucleus while accounting for the embryo curvature (see Figure 5A for illustration).

16. Download the custom python package “sass” (**S**imple **A**ssignment of **S**pots to **S**urfaces), which links the identified transcription foci to their respective nuclei. The package containing all scripts can be downloaded here: https://github.com/TMinchington/sass
  a. Make sure that python 3 (or Anaconda3) is installed on the computer.
  b. Using the Anaconda Prompt, navigate to the folder that contains the sass.py file (alternatively, drag and drop the sass.py file into the command prompt).
  c. Type: python sass.py [drag and drop the parent folder containing the subfolder with the exported Imaris statistics] and run the analysis (Figure 6, step 2).
    i. If the optional step of mitosis correction was skipped, sass.py will show this message before finishing “No key time file detected. Would you like to stop and create this file now? (y/n).” Answer with “n” in the command line.
  d. The output folder is generated in the subfolder containing the Imaris statistics. The folder “time data” contains the finished analysis files.
    i. The image mid_line_expression.png shows a summary image of nuclei with active transcription (green), nuclei without transcription (blue) and the midline of the expression domain (Figure 6, step 3).
      ***Note:*** Nuclei in Figure 6, step 3 are identified as inactive (blue) because either no transcription sites were identified in step 13 or because the nucleus was excluded as it is not present in the field of view during the full transcription period (see the Note below).
    ii. The image assignments.png file shows the percentage of nuclei that were assigned 1 spot (blue line), 2 spots (orange line) or 3 spots (green line) for each time frame (X-axis) (Figure 6, step 4). This graph can be used as a visual aid to assess the segmentation success. Optimally, nuclei should only have been assigned 1 spot (see troubleshooting 6).
    iii. The file “mid_line.txt” contains the coordinates for the expression domain midline.
    iv. The file “backed_down-hc.txt” contains the tracked nuclei and transcription spots (identify by nuclear ID in the column “nuc” and spot ID in the column “spot”) and their 3D positions (columns “nucx/y/z” and “spotsx/y/z”). The nuclear X/Y/Z positions can be used to determine the nuclear position along the embryo’s AP and DV axis. The fluorescence value, background corrected value and the fluorescence intensity can be found in the columns “value,” “corrected_val” and “variable” respectively. Make sure to filter for the MCP-GFP channel in the column “channel” for further analysis. The distance to the midline can be found in the column “dis_mid” and is computed in μm. Imaging time can be found in the column “time” and is calculated in min (Figure 6, step 5). If the optional step of mitosis adjustment was included, the adjusted time can be found in column “time_shift” (Figure 6, step 5).
  e. Using the nuclear ID, full traces of transcriptional activity can be plotted or used for further analysis. If multiple spots are assigned to a single nucleus see troubleshooting 6.
    ***Note:*** The default microscopy time interval setting is 20 s in the python package. This can be overwritten by running the scripts with the following modification: python sass.py **--i [time interval in sec]** [folder path to analyse]*.* A time interval of 60 s will result in full integers, as the time interval is displayed in min. For example: python sass.py --i 60 “D:\Carol\Methods paper\Analysis.”
    ***Note:*** For downstream analysis, we recommend using “Intensity Sum” fluorescence values as these contain the full fluorescence detected in 3D.
    ***Note:*** The file “backed_down-hc.txt” only contains a subset of nuclei. Contained in this list are nuclei that were present in the first and last time frame during gene transcription. Nuclei that entered or exited the field of view are excluded. Full data can be found in the “backed_down.txt” file.
    **CRITICAL:** The quality of the analyzed data should be inspected before continuing with downstream analysis. The assignments.png file in the analysis folder can be used as an indicator for successful nuclear tracking and spot identification (less than 5% of nuclear traces have more than 1 spot assigned). If this value is found to be above 5% this could be due to false-positive spots or gaps in nuclear tracks (see troubleshooting 6)**.** We also recommend plotting the mean fluorescence intensity and transcription onset time of all analyzed nuclei as well as a few individual nuclear traces to visualize obvious problems with the dataset (in comparison with other biological replicates) before progressing with downstream analysis.

## Expected outcomes

**Figure 7:**
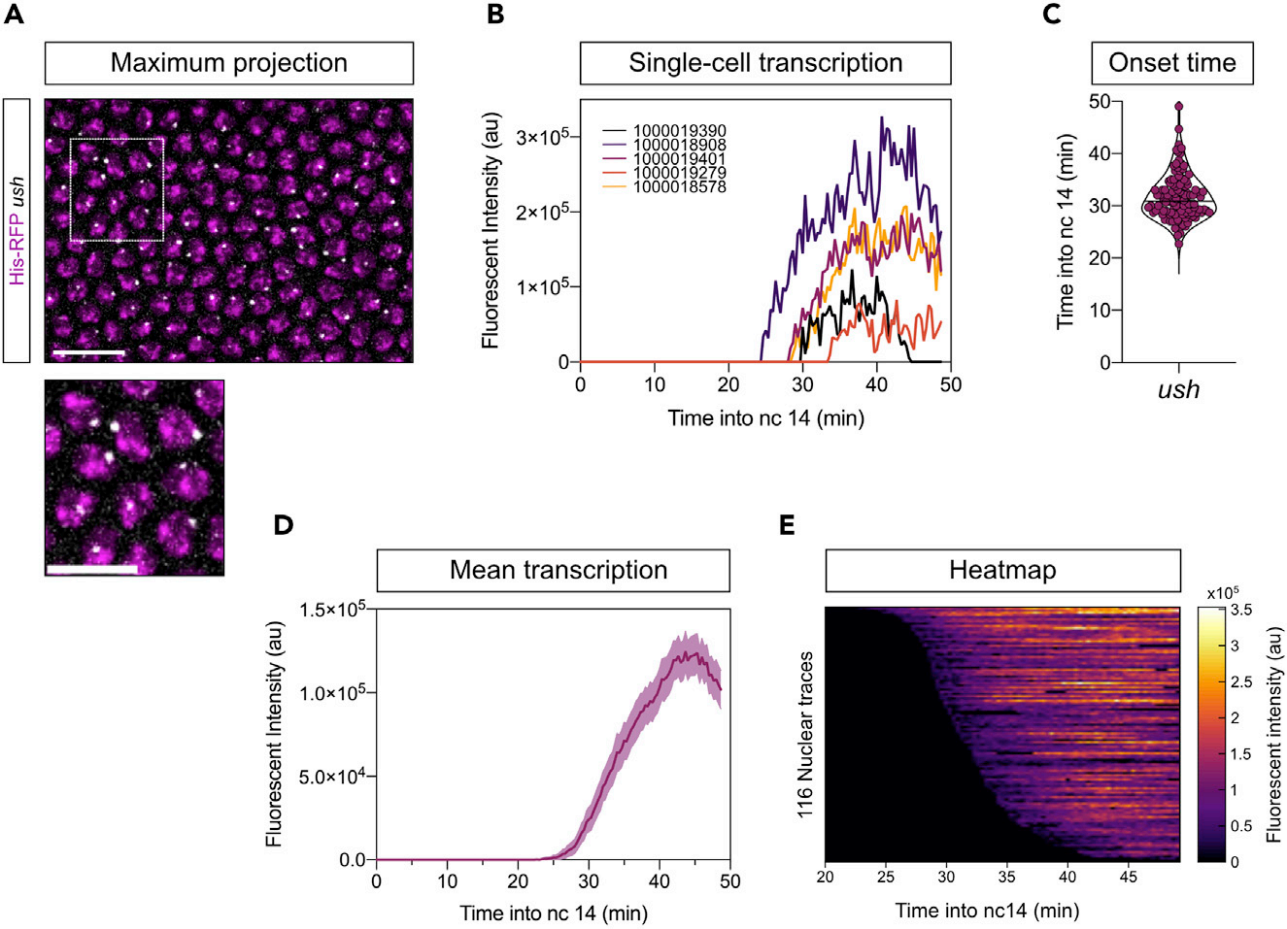
Analysis of 24xMS2-*ush* transcription profiles in the *Drosophila* embryo (A) Still from a cropped time lapse movie maximum projected showing active *ush* transcription. Transcription is shown by MCP-GFP fluorescence (gray) and nuclei are shown by His-RFP (magenta). The enlarged region (lower panel) shows one active transcription site per nucleus. (B) Representative transcription traces of *ush* over developmental time. Nuclear IDs are shown for identification of the traces in the example dataset. (C) Transcription onset times of *ush*. (D) Mean *ush* fluorescence over time combines the fluorescence values for all nuclei. (E) Heatmap of single-cell fluorescence- traces, sorted according to transcription onset (scale as indicated). Scale bar, 15 μm (A, top) and 10 μm (A, bottom). Median (C) and mean ± 95% confidence intervals (D). (C)–(E) contain data from 116 nuclei that are tracked and present in the example dataset.

Tagging genomic regions of interest (either endogenous loci or transgenes) with MS2 stem-loops results in a bright fluorescent signal representing nascent transcription when imaged using a confocal microscope (Figure 7A). Visualization of the single-cell transcription profiles of the Bone Morphogenetic Protein (BMP) signaling pathway target genes *u-shaped* (*ush*) and *hindsight* identified significant differences in transcription kinetics based on the level of signaling that cells are exposed to (Hoppe et al., 2020). These genes are expressed during nc14 and are important for early embryonic development (Ashe et al., 2000).

The example dataset included for this protocol shows *ush* transcription during nc14 (Figure 7A). Using the semi-automated image analysis pipeline outlined here, the nascent mRNA signal is extracted and single-cell fluorescent traces can be displayed, highlighting differences in transcription between cells (Figure 7B). Additionally, the transcription onset time of individual cells can be determined placing the median onset time of *ush* transcription at 31 min into nc14 in the example dataset (Figure 7C). As well as analyzing single-cell transcription dynamics, nuclei can be grouped and the mean transcriptional activity of *ush* determined (Figure 7D). The differences in single-cell transcription profiles and the transcriptional onset time can be visualized using heatmaps to investigate spatial and temporal transcription dynamics (Figure 7E).

The fluorescence emitted by a nascent transcription site is assumed to be directly proportional to the number of mRNA molecules that are produced. Due to this relationship, the fluorescent signal can be converted into the number of elongating Pol II molecules using a conversion based on single molecule fluorescent *in situ* hybridization (smFISH) data. Descriptions of how the MCP-GFP fluorescence can be converted into Pol II number can be found in these studies (Garcia et al., 2013; Hoppe et al., 2020; Lammers et al., 2020).

***Note:*** We would recommend analyzing at least three biological replicates per genotype and a minimum of 100 nuclear traces per replicate (embryo). To achieve robust modeling results using the memory adjusted Hidden Markov Model (Bowles et al., 2020; Lammers et al., 2020) (see quantification and statistical analysis) we recommend using a minimum of 1,000 data points (for example 50 nuclear traces with a transcription signal in 20 time frames).

## Quantification and statistical analysis

To analyze transcription dynamics in greater detail, a memory adjusted Hidden Markov Model can be used to extract transcriptional burst kinetics (Bowles et al., 2020; Hoppe et al., 2020; Lammers et al., 2020). Using this computational approach, the underlying promoter states corresponding to each time frame in the time-lapse data can be inferred and transcription parameters can be calculated. This approach assumes a two-state promoter model, where the promoter transitions between ON and OFF states with specific rates *k*_*on*_ and *k*_*off*_ (Lammers et al., 2020; Zoller et al., 2018).

By calculating transcription parameters, the transcriptional response to, for example, signaling levels can be quantified and compared between genotypes or at different positions within the embryo. A full analysis of how single cells decode the BMP signal through modulation of target gene transcription can be found in Hoppe et al., 2020. Detailed information to implement downstream analysis using the memory adjusted Hidden Markov Model can be found in Lammers et al., 2020 and Bowles et al., 2020, which can also be used to infer transcription parameters at single-cell resolution (Bowles et al., 2020).

## Limitations

Although the MS2/MCP live imaging system offers a robust tool to image gene transcription, it has some limitations. Firstly, transcription from weak promoters will be hard to detect and quantify. Due to the inherent background of unbound MCP-GFP protein in the nucleus, a weak transcription signal will result in a low signal-to-noise ratio and therefore be hard to detect.

Secondly, most published *Drosophila* lines expressing the MCP fusion protein use a monomer MCP molecule. However, *in vitro* experiments showed varying degrees of saturation of the MS2 stem-loops with MCP-GFP, particularly at lower MCP-GFP concentrations (Fusco et al., 2003; Wu et al., 2012). Hence, the amount of nascent fluorescence may not be exactly proportional to the mRNA number produced, as is assumed for analysis. The low occupancy was increased *in vitro* by expressing MCP as a single chain tandem dimer (Wu et al. 2012). Fly lines using an MCP tandem dimer are predicted to promote uniform binding to MS2 loops even at lower concentrations of the MCP-fluorophore fusion protein. Fly lines are available with NLS-2xMCP-TagRFP-T (Halstead et al., 2015), 2xMCP-2xRFP and 2xMCP-2xmRuby3 (Ashe lab unpublished, available on request from the lead contact).

## Troubleshooting

### Problem 1

Embryos slide during the live imaging experiment or show mitotic division arrest.

### Potential solution

Both observations can be caused by problems with the mounting of the embryos onto the heptane glue coated coverslip or if the Lumox imaging dish membrane is too flexible.

If embryos were not attached properly to the heptane glue covered coverslip, they might slide during the live-cell imaging experiment. This could be caused by the heptane glue being too dilute. Add new double-sided tape to the heptane glue bottle. Once the glue has been dissolved, the heptane/glue solution should become more viscous. Alternatively, the embryos were not pressed against the coverslip sufficiently. After dropping the coverslip onto the embryos, make sure that the embryo is flattened against the coverslip by carefully pressing onto the coverslip with tweezers. Sliding or other movement can also be caused if the membrane of the Lumox imaging dish is too flexible. When reusing the imaging dishes, the membrane can become damaged from removing coverslip bridges from the previous experiments. Make sure to not reuse the dishes more than 2–3 times and if in doubt use a fresh dish.

On occasion, we have observed that nuclei can arrest during mitotic cleavage cycles in parts or the full embryo. We have identified one reason causing this arrest to be too much pressure of the coverslip against the embryo. Be careful not to attach the embryo too forcefully to the coverslip.

### Problem 2

Photobleaching is observed during the time-lapse experiment.

### Potential solution

If photobleaching is observed, decrease the laser power and/or reduce the sampling frequency.

### Problem 3

Unable to distinguish MCP-GFP background fluorescence from real MS2/MCP fluorescent signal or very low MS2/MCP fluorescent signal.

### Potential solution

Try to increase the laser power and use very sensitive detectors. Increase the signal-to-noise ratio by using flies that are heterozygous for the MCP-GFP transgene. This reduces the amount of unbound MCP-GFP that is maternally deposited. Inserting the MS2 stem-loops into the 5′ UTR of a gene will maximize the fluorescence as the stem-loops will be bound by MCP-GFP for longer.

Another approach to increase the signal-to-noise ratio is to increase the number of MS2 stem-loop repeats that are inserted into the endogenous gene locus, for example to 128 copies (Tantale et al., 2016). More MS2 stem-loops will bind more fluorophores, which will increase the overall fluorescent signal aiding detection. However, the insertion of more MS2 stem-loops will increase the size of the DNA sequence inserted, potentially leading to artifacts in gene expression regulation.

### Problem 4

Dim transcription sites are not detected by Imaris.

### Potential solution

If transcription foci are very dim they might not be identified and need to be added manually. This can be done in the editing tab with the pointer in the “select” mode. Hover over the transcription spot, press shift, and left click to add a sphere. Adjust the sphere size to match the other added spots. Depending on the number of transcription sites that need to be added manually, correction can be very time consuming. If the number of spots that need to be corrected causes a significant increase in analysis time, consider analyzing a smaller region of interest or changing the microscopy settings to enhance the quality of raw data (higher resolution/ higher magnification/ increased laser power).

### Problem 5

Many false-positive spots are detected by Imaris.

### Potential solution

If many false-positive spots are detected by the software, try to break up spot detection into smaller time intervals. Assign spots to 5–10 time frames at a time and merge spot files afterwards. Remove remaining false-positive spots manually by highlighting the spot and deleting it in the editing tab. If the time it takes to manually correct the dataset increases the analysis time by hours, consider analyzing a smaller subset of nuclei or changing the microscopy settings (see Problem 4).

### Problem 6

Multiple transcription spots have been assigned to one nucleus.

### Potential solution

There are different reasons for wrongly assigned spots. Firstly, if false-positive spots (that are not transcription spots) were identified during spot creation, they will have been assigned to the closest nucleus, which might show active transcription. False-positive spots can often be identified by a very low fluorescent value (close to background values) recorded for this spot.

Secondly, a mistake could have been made during the segmentation of nuclei. If two nuclei are very close to each other, they can be fused into one during nuclei detection. In the subsequent tracking one nuclear trace would then be missing a time point (where the nucleus was fused to its neighbor). If a transcription site was detected in the time frame with the missing nucleus, it will be wrongly assigned to a different nucleus. Alternatively, if two nuclei come into too close proximity it is possible that a transcription site was wrongly assigned to a neighbor based on a closer proximity in three dimensions.

Finally, wrongly assigned transcription foci can occur at the edge of the time-lapse video. If nuclei are entering or exiting the field of view they might not have been detected during nuclear segmentation as they can appear smaller with less fluorescent intensity when they are partially cut off. If the transcription sites of these nuclei were detected, they will have been wrongly assigned to a neighboring nucleus. In our experience it works best to exclude nuclei and their respective transcription sites that start off on the edge of the field of view.

To check the percentage of nuclei that have 2 or more transcription foci assigned to them, check the assignments.png file in the analysis folder (Figure 6, step 4).

## Resource availability

### Lead contact

Further information and requests for resources and reagents should be directed to and will be fulfilled by the lead contact, Hilary L. Ashe (hilary.ashe@manchester.ac.uk).

### Materials availability

The His-RFP; MCP-GFP fly line used in this protocol is available from the Bloomington *Drosophila* Stock Center and the *24xMS2-ush* line is available upon request from the lead contact.

### Data and code availability

Two example datasets, a beginner and full dataset, containing Imaris files and image analysis files are deposited at Mendeley Data https://doi.org/10.17632/jv9fjpn9cg.1. The custom analysis code “sass” that links Imaris output files is available at GitHub (https://github.com/TMinchington/sass, RRID:SCR_018797). The modeling software mentioned in the “quantification and statistical analysis” section is described in Bowles et al. (2020).
